# Pres-FINAL-2009: Pediatric rheumatology practitioners experience with biologics in juvenile dermatomyositis: survey results

**DOI:** 10.1186/1546-0096-11-S2-P22

**Published:** 2013-12-05

**Authors:** A Patwardhan, K Rouster-Stevens, C Spencer

**Affiliations:** 1 Pediatric Rheumatology, University of Misouri School of Medicine, Columbia, MO, USA; 2 Pediatric Rheumatology, Ohio State University, Columbus, OH, USA; 3 Pediatric Rheumatology, University of Emroy, Atlanta, USA

## Introduction

Biologic therapy is increasingly prescribed in rheumatologic disorders. Juvenile dermatomyositis (JDM), the most common inflammatory myopathy in children, can be challenging to manage in a subset of patients. There are multiple reports of cytokine involvement in JDM. There is a paucity of information regarding the use of biologics for JDM among pediatric rheumatology practitioners, and only one clinical trial investigating a biologic in JDM.

## Objectives

The purpose of the study is to examine pediatric rheumatology (PR) experience in North America and Canada with biologic therapy in children with JDM.

## Methods

The Childhood Arthritis and Rheumatology Research Alliance JDM Subcommittee on Biologics developed a 15-question on-line survey. Of the 231 pediatric rheumatology practitioners contacted, 105 (45%) participated between 2/17-3/20/2012.

## Results

Over half (57%) of the respondents currently managed 1-10 patients with JDM; 10% of respondents reported ≥20 patients with JDM in their practice. Sixty-one percent of respondents had used biologics in patients with JDM, with 32%, 5%, and 4% prescribing rituximab, etanercept and infliximab, respectively; 17% had prescribed more than one biologic. The majority of respondents (89%) had used biologics in combination with other therapies, while 11% had used biologics as monotherapy in JDM. The biologics used by the respondents were, rituximab, infliximab, etanercept and anakinra and abatacept. Among the respondents that used biologics, uncontrolled disease was the primary rationale for prescribing this medication. Over half of respondents used biologics after the patients failed other therapies; 11% of respondents used biologics for systemic (internal organ) involvement and 15% had used biologics for severe ulcerative disease. Seventy-three percent of respondents that used biologics noted improvement, while 10% reported worsening disease. Over half (53%) of respondents that used biologics noted improvement in calcinosis, while 64% reported side effects (common and uncommon). Among the respondents that had not used biologics (39%) in JDM, 88% would use this therapy if the opportunity arose; nearly half (47%) of these respondents had not used biologics because of uncertainty regarding effectiveness in JDM. Seventy percent of practitioners recommended that biologics be formally studied in patients with JDM; 24% of respondents were unsure and 6% felt biologics should not be studied in patients with JDM.

## Conclusion

Several PR have used biologics in the management of pediatric patients with JDM. Among respondants that have not used biologics in this patient population, most would be interested in prescribing biologics. This survey supports the rationale for considering clinical trials and consensus protocols to elucidate the safety and effectiveness of biologics in children with JDM. Further information will be gathered by the CARRA JDM Subcommittee on Biologics through second survey to prioritize specific medications for investigation.

## Disclosure of interest

None declared.

